# Combined effect of inflammation and malnutrition for long-term prognosis in patients with acute coronary syndrome undergoing percutaneous coronary intervention: a cohort study

**DOI:** 10.1186/s12872-024-03951-7

**Published:** 2024-06-17

**Authors:** Yang Yuxiu, Xiaoteng Ma, Fei Gao, Tao Liu, Jianping Deng, Zhijian Wang

**Affiliations:** 1 Department of Cardiology, Center for Coronary Artery Disease, Beijing Anzhen Nanchong Hospital, Capital Medical University, 2 Anzhen Road, Chaoyang District, Beijing, 100037 China; 2 Department of Cardiology, Center for Coronary Artery Disease, Beijing Anzhen Nanchong Hospital, Capital Medical University, Nanchong, Sichuan 637000 China

**Keywords:** Inflammation, Malnutrition, Acute coronary syndrome, Nutritional risk index

## Abstract

**Background:**

Inflammation is a key driver of atherosclerotic diseases and is often accompanied by disease-related malnutrition. However, the long-term burden of dysregulated inflammation with superimposed undernutrition in patients with acute coronary syndrome (ACS) remains unclear. This study sought to investigate the double burden and interplay of inflammation and malnutrition in patients with ACS undergoing percutaneous Coronary Intervention (PCI).

**Methods:**

We retrospectively included 1,743 ACS patients undergoing PCI from June 2016 through November 2017 and grouped them according to their baseline nutritional and inflammatory status. Malnutrition was determined using the nutritional risk index (NRI) with a score lower than 100 and a high-inflamed condition defined as hs-CRP over 2 mg/L. The primary outcome was major adverse cardiovascular events (MACEs), compositing of cardiac mortality, non-fatal myocardial infarction, non-fatal stroke, and unplanned revascularization. Long-term outcomes were examined using the Kaplan-Meier method and compared with the log-rank test. Multivariable Cox proportional hazards regression analysis was applied to adjust for confounding. The reclassification index (NRI)/integrated discrimination index (IDI) statistics estimated the incremental prognostic impact of NRI and hs-CRP in addition to the Global Registry of Acute Coronary Events (GRACE) risk score.

**Results:**

During a median follow-up of 30 months (ranges 30–36 months), 351 (20.1%) MACEs occurred. Compared with the nourished and uninflamed group, the malnourished and high-inflamed group displayed a significantly increased risk of MACEs with an adjusted hazard ratio of 2.446 (95% CI: 1.464–4.089; *P* < 0.001). The prognostic implications of NRI were influenced by patients’ baseline inflammatory status, as it was only associated with MACEs among those high-inflamed (*P* for interaction = 0.005). Incorporating NRI and hs-CRP into the GRACE risk score significantly improved its predictive ability for MACEs (NRI: 0.210, *P* < 0.001; integrated discrimination index; IDI: 0.010, *P* < 0.001) and cardiac death (NRI: 0.666, *P* < 0.001; IDI: 0.023, *P* = 0.002).

**Conclusions:**

Among patients with ACS undergoing PCI, the double burden of inflammation and malnutrition signifies poorer outcomes. Their prognostic implications may be amplified by each other and jointly improve the GRACE risk score’s risk prediction performance.

**Supplementary Information:**

The online version contains supplementary material available at 10.1186/s12872-024-03951-7.

## Background

Acute coronary syndrome (ACS) is still among the leading causes of global mortality and morbidity [1]. Studies in recent years have raised a particular concern that malnutrition is not insubstantial in ACS patients, even among those obese [2, 3]. Malnutrition fosters an increased burden through the vicious cycle of sarcopenia, cardiac dysfunction, and cardiogenic cachexia in cardiovascular diseases (CVD) [4, 5], and it’s also a modifiable risk factor on which physicians could act [6]. The nutritional risk index (NRI) has been commonly used as a nutritional screening tool in these years’ studies for its simplicity, accessibility, and discrimination abilities [7–10]. As a systemic and dynamic pathological process, inflammation plays multiple maladaptive roles contributing to the progression and destabilization of ACS, including atherogenesis, plaque evolution, thrombogenesis, as well as myocardial damage and repair [11, 12]. However, there is still an unmet need to take cognizance of the double burden of malnutrition and inflammation on ACS patients and identify those who would benefit more from proactive anti-inflammatory therapy.

Dysregulated inflammation with superimposed undernutrition may constitute a significant yet relatively concealed threat in ACS patients, which needs additional attention and integrated responses. Here, we aimed to explore the combined effects and interplay of inflammation and malnutrition on outcomes in ACS patients to provide valuable information as we move toward more individualized CVD care.

## Methods

### Study design and population

In this single-center, retrospective observational cohort, patients presenting with ACS undergoing percutaneous coronary intervention (PCI) between June 2016 and November 2017 at Beijing Anzhen Hospital were consecutively included. Patients with missing data on height, weight, serum albumin, hs-CRP, and a known history of coronary artery bypass grafting were excluded. Baseline demographic data, previous medical history, clinical presentations, laboratory and echocardiographic results, angiographic and procedural characteristics, and medications were collected from the electronic medical records. This study followed the Helsinki Declaration of Human Rights and was approved by the institutional review board (IRB) of Beijing Anzhen Hospital (IRB number: 2016034x). The IRB waived the need for written patient consent as this study involved a retrospective analysis of clinically acquired data.

### Definitions

We dichotomized the inflammatory status of ACS populations based on enrollment hs-CRP measurements of 2 mg/L or greater (high-inflamed) vs. less than 2 mg/L (uninflamed), consistent with the current consensus [13, 14]. Nutritional status was graded using the NRI, which was calculated using [1.519 × serum albumin (g/L)] + 41.7 × (current weight/ideal weight)] for patients under the age of 65 and [1. 489×serum albumin (g/L)] + 41.7 × (current weight/ideal weight)] for those over 65, the latter formula is also known as the geriatric NRI (GNRI) [15]; the ideal body weight was considered using the Lorenz formula, that is, [height (cm) -100 - ([height (cm) − 150]/4)] for men, and [height (cm)-100-([height (cm)-150]/2.5)] for women [16]; malnutrition was considered as a dichotomous variable as scores of NRI or GNRI lower than 100 considered malnutrition [15]. Acute coronary syndrome and its components were determined by clinical diagnosis at discharge extracted from the electronic medical record. Anemia was hemoglobin lower than 120 g/L for men and lower than 110 g/L for women [17]. Complete revascularization was defined as treating all coronary artery segments > 1.5 mm in diameter and ≥ 50% diameter stenosis in the same procedure [18], which was determined according to the final coronary angiography reports.

### Outcomes

The primary outcome was major adverse cardiovascular events (MACEs), defined as the composite proportion of cardiac mortality, non-fatal MI, non-fatal stroke, and unplanned revascularization. Cardiac mortality includes deaths that result from an AMI, sudden cardiac death, heart failure, and cardiac procedures. An unplanned revascularization was defined as a revascularization procedure that was not planned at the time of the index AMI admission and that was not performed in the setting of a recurrent AMI (to avoid examining the clinical impact of recurrent AMIs). The study index date was the admission date, and complete follow-up was until any MACE occurred or December 2020, whichever came first. Patients were followed up every six months by telephone or through available medical records.

### Statistical analysis

Categorical variables were summarized as frequencies and percentages; numerical variables were summarized as the mean (standard deviation, SD) or median (interquartile range, IQR), depending on the data distribution. Categorical variables were compared with the χ² test, and continuous variables with Mann-Whitney non-parametric tests. Receiver-operating characteristic curve (ROC) analyses were performed to estimate the discriminative abilities of the nutritional scores and systemic inflammatory indicators on MACEs. The associations between the variables and the binary study outcomes were assessed using multivariable Cox proportional hazards regression models with a hazard ratio (HR) and 95% confidence interval (CI) in the total population and by stratified groups. Besides, the interactions between malnutrition and inflammation were tested by conducting likelihood ratio tests.

Potential covariates were identified by reviewing existing literature and clinician consensus. The following variables were extracted from the baseline assessment data: demographic factors (age and sex); clinical characteristics (heart rate, systolic blood pressure, diastolic blood pressure, hypertension, diabetes, peripheral artery disease, old myocardial infarction, prior PCI, prior stroke, anemia, total cholesterol [TC], low-density lipoprotein cholesterol [LDL-C], creatinine clearance [CrCl], fast plasma glucose [FBG], glycated hemoglobin A1c [HbA1C], left ventricular systolic dysfunction [LVSD], multivessel coronary artery disease, SYNTAX [Synergy Between PCI With Taxus and CABG] score, and complete revascularization); and discharge pharmacotherapy (dual antiplatelet therapy [DAPT], statin, and beta-blockers). Kaplan-Meier survival probability estimates were calculated from the date of PCI up to the total available follow-up at three years, stratified according to nutritional and inflammatory status, and assessed with the log-rank test. The concordance index, net reclassification improvement, and integrated discrimination improvement statistical analyses were performed to evaluate the incremental value of the hs-CRP or NRI in addition to the GRACE (Global Registry of Acute Coronary Events) risk model. A two-sided P-value of less than 0.05 was considered to indicate statistical significance. All analyses were done with R software (version 3.5.0, Austria) and Prism software (version 5.0, USA).

## Results

**Fig. 1:**
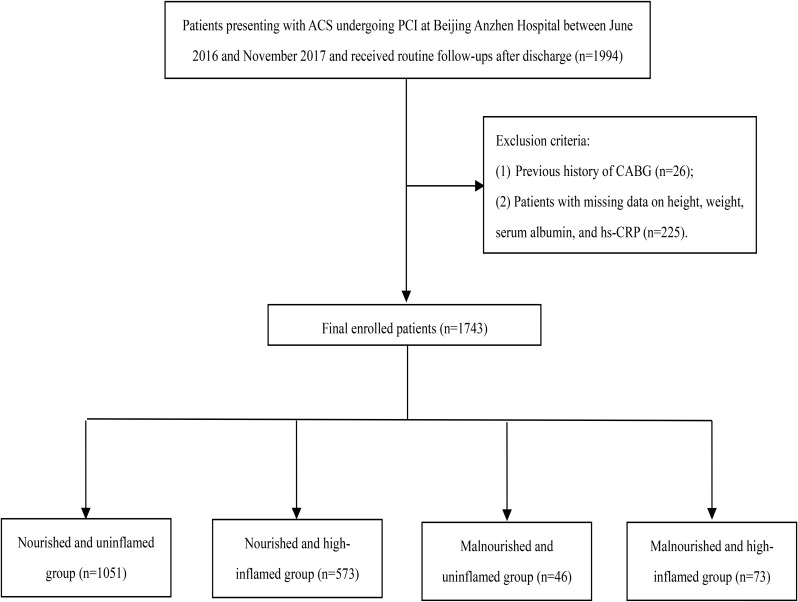
Flow chart of the study population enrollment and stratification *Abbreviations* ACS, acute coronary syndrome; CABG, coronary angioplasty bypass grafting; CRP, C-reactive protein; PCI, percutaneous coronary intervention

A total of 1,743 ACS patients treated with PCI who met the enrollment criteria were included (251 patients without complete informative data were excluded), with an average age of 60 years, of whom 76.5% were male. Patients were stratified into four phenotypes according to their NRI and hs-CRP categories: nourished and uninflamed group (*n* = 1051), nourished and high-inflamed group (*n* = 573), malnourished and uninflamed group (*n* = 46), as well as malnourished and high-inflamed group (*n* = 73) (Fig. 1). Over a median follow-up of 30 months (IQR, 30–36months), 351 (20.1%) MACEs occurred, including 37 (2.1%) cardiac deaths, 43 (2.5%) non-fatal MI, 22 (1.3%) non-fatal stroke, and 249 (14.3%) unplanned coronary revascularization. Compared with the other groups, malnourished and highly inflamed patients were more prone to complicated diabetes, anemia, and renal dysfunction, presenting with larger proportions of acute myocardial infarction (AMI), displaying worse cardiac function and more complex coronary lesions; they were also more often to be prescribed with furosemide on discharge. After 30 months of follow-up, the malnourished and high-inflamed group showed the highest rates of MACEs (41.1%), cardiac death (12.3%), non-fatal MI (4.1%), and unplanned revascularization (24.7%) among the four phenotypes (Table 1).

**Table 1:** Baseline clinical characteristics and outcomes of patients stratified by the nutritional and inflammatory statuses

Variable	Total population (*n* = 1743)	Nourished and uninflamed (*n* = 1051)	Nourished and high-inflamed (*n* = 573)	Malnourished and uninflamed (*n* = 46)	Malnourished and high-inflamed (*n* = 73)	*P* value ^a^
**Demography and anthropometric data**						
Age, years	60 (53, 67)	61 (53, 66)	61 (52, 68)	63 (58, 69)	62 (54, 73)	0.028
Sex, male, n (%)	1333 (76.5)	824 (78.4)	428 (74.7)	29 (63.0)	52 (71.2)	0.030
Body mass index, kg/m^2^	25.2 (23.6, 27.6)	25.3 (23.7, 27.5)	26.0 (24.0, 28.3)	21.6 (20.0, 23.3)	22.6 (21.1, 23.8)	< 0.001
Heart rate, bpm	68 (62, 75)	67 (61, 73)	69 (63, 76)	70 (62, 74)	71 (66, 80)	< 0.001
Systolic blood pressure, mmHg	130 (120, 140)	130 (120, 140)	130 (120, 140)	100 (118, 133)	120 (110, 130)	< 0.001
Diastolic blood pressure, mmHg	76 (70, 81)	78 (70, 82)	74 (69, 80)	71 (66, 77)	68 (64, 73)	< 0.001
Family history of CAD, n (%)	554 (31.5)	335 (31.9)	192 (33.5)	6 (13.0)	21 (28.8)	0.035
**CV risk factors and prior CV disease**						
Hypertension, n (%)	1112 (63.8)	377 (64.4)	373 (65.1)	16 (34.8)	46 (63.0)	< 0.001
Diabetes, n (%)	805 (46.2)	474 (45.1)	278 (48.5)	16 (34.8)	37 (50.7)	0.191
Hypercholesterolemia, n (%)	1396 (80.1)	809 (77.0)	494 (86.2)	32 (69.6)	61 (83.6)	< 0.001
Current smoking, n (%)	1025 (58.8)	617 (58.7)	345 (60.2)	24 (52.2)	39 (53.4)	0.539
Old myocardial infarction, n (%)	333 (19.1)	201 (19.1)	105 (18.3)	12 (26.1)	15 (20.5)	0.621
Prior PCI, n (%)	343 (19.7)	223 (21.2)	100 (17.5)	13 (28.3)	7 (9.6)	0.017
Prior Cardiac arrest, n (%)	2 (0.1)	0 (0)	2 (0.3)	0 (0)	0 (0)	0.240
Atrial fibrillation/flutter, n (%)	74 (4.2)	45 (4.3)	24 (4.2)	2 (4.3)	3 (4.1)	1.000
Prior stroke, n (%)	102 (5.9)	53 (5.0)	44 (7.7)	3 (6.5)	2 (2.7)	0.103
Peripheral artery disease, n (%)	183 (10.5)	91 (8.7)	78 (13.6)	4 (8.7)	10 (13.7)	0.013
**Comorbidity**						
Anemia, n (%)	57 (3.3)	17 (1.6)	25 (4.4)	3 (6.5)	12 (16.4)	< 0.001
COPD, n (%)	26 (1.5)	12 (1.1)	12 (2.1)	0 (0)	2 (2.7)	0.273
Cancer history, n (%)	13 (0.7)	8 (0.8)	4 (0.7)	0 (0)	1 (1.4)	1.000
**ACS presentation**						
Type of ACS, n (%)						
STEMI	229 (13.1)	90 (8.6)	110 (19.2)	4 (8.7)	25 (34.2)	< 0.001
NSTEMI	227 (13.0)	105 (10.0)	99 (17.3)	8 (17.4)	15 (20.5)	< 0.001
Unstable angina	1287 (73.8)	856 (81.4)	364 (63.5)	34 (73.9)	33 (45.2)	< 0.001
Killip class > 1, n (%)	67 (3.8)	22 (2.1)	26 (4.5)	4 (8.7)	15 (20.5)	< 0.001
The GRACE risk score, points	98 (82, 114)	94 (92, 108)	99 (82, 119)	105 (91, 118)	120 (102, 155)	< 0.001
High risk at six months, n (%)	356 (20.4)	158 (15.0)	150 (26.2)	11 (23.9)	37 (50.7)	< 0.001
**Laboratory data and Echocardiographic data**						
White blood cell, 10^9^/L	6.3 (6.3, 7.5)	6.0 (5.2, 7.0)	7.1 (6.0, 8.1)	6.1 (5.0, 7.0)	7.3 (6.2, 9.6)	< 0.001
Neutrophil-lymphocyte ratio	2.2 (1.7, 3.0)	1.0 (0, 1.0)	2.4 (1.9, 3.2)	2.3 (1.9, 3.1)	3.1 (2.3, 4.2)	< 0.001
High-sensitivity C-reactive protein, mg/L	1.7 (0.7, 3.5)	0.78 (0.4, 1.3)	4.6 (3.1, 8.9)	0.7 (0.4, 1.3)	9.5 (4.8, 18.5)	< 0.001
Triglycerides, mmol/L	1.5 (1.0, 2.1)	1.4 (1.0, 2.0)	1.6 (1.2, 2.2)	1.1 (0.7, 1.7)	1.3 (1.0, 1.8)	< 0.001
Total cholesterol, mmol/L	4.0 (3.4, 4.8)	4.0 (3.4, 4.7)	4.2 (3.6, 5.0)	3.6 (3.1, 4.2)	3.7 (3.4, 4.5)	< 0.001
LDL-C, mmol/L	2.3 (1.8, 3.0)	2.3 (1.8, 2.8)	2.6 (2.0, 3.0)	1.9 (1.6, 2.4)	2.2 (1.8, 3.0)	< 0.001
HDL-C, mmol/L	1.0 (0.9, 1.2)	1.0 (0.9, 1.2)	1.0 (0.8, 1.1)	1.0 (0.9, 1.3)	0.9 (0.8, 1.1)	< 0.001
B-type natriuretic peptide, pg/ml	37 (21, 86)	33 (20, 67)	33 (20, 67)	40 (24, 90)	197 (52, 197)	< 0.001
Albumin, g/L	42 (40, 45)	43 (40, 45)	45 (23, 118)	37 (36, 39)	36 (34, 38)	< 0.001
Creatinine clearance rate, ml/min	96.7 (78.8, 116.7)	98.1 (81.2, 117.6)	97.8 (78.5, 120.0)	83.4 (67.1, 97.5)	80.1 (66.1, 92.9)	< 0.001
Fasting plasma glucose, mmol/L	5.8 (5.2, 7.0)	5.8 (5.2, 6.8)	6.0 (5.3, 7.2)	5.6 (4.9, 6.7)	6.1 (5.3, 7.4)	0.007
Glycated hemoglobin A1c, %	6.1 (5.6, 7.1)	6.0 (5.5, 7.0)	6.3 (5.6, 7.3)	6.0 (5.5, 6.5)	6.3 (5.7, 7.4)	0.002
LVSD (EF < 50%), n (%)	106 (6.1)	42 (4.0)	45 (7.9)	6 (13.0)	13 (17.8)	< 0.001
**Nutrition risk index**	111.5 (105.4, 117.0)	112.7 (107.9, 117.4)	112.2 (106.1, 117.5)	98.2 (96.5, 99.4)	97.2 (95.0, 98.6)	< 0.001
**Angiographic data**						
Left main coronary artery lesion, n (%)	136 (7.8)	71 (6.8)	55 (9.6)	4 (8.7)	6 (8.2)	0.213
Multivessel coronary artery disease, n (%)	1457 (83.6)	866 (82.4)	493 (86.0)	35 (76.1)	63 (86.3)	0.116
Chronic total occlusion, n (%)	367 (21.1)	208 (19.8)	141 (24.6)	5 (10.9)	13 (17.8)	0.036
SYNTAX score	20 [13, 28]	19 [12, 27]	22 [15, 29]	21 [11, 30]	26 [17, 34]	< 0.001
Complete revascularization, n (%)	1069 (61.3)	687 (65.4)	312 (54.5)	31 (67.4)	39 (53.4)	< 0.001
Percutaneous transfemoral route, n (%)	27 (1.5)	21 (2.0)	4 (0.7)	0 (0)	2 (2.7)	0.122
**Medical therapy**						
Pre-admission, n (%)						
Antiplatelet agents	1272 (73.0)	781 (74.3)	403 (70.3)	37 (80.4)	51 (69.9)	0.205
Statin	1252 (71.8)	772 (73.5)	389 (67.9)	39 (84.8)	52 (71.2)	0.021
Discharge, n (%)						
Dual antiplatelet therapy	1727 (99.1)	1047 (99.6)	565 (98.6)	45 (97.8)	70 (95.9)	0.013
Aspirin plus clopidogrel	1587 (91.0)	968 (92.1)	508 (88.7)	42 (91.3)	69 (94.5)	0.086
Aspirin plus ticagrelor	140 (8.0)	79 (7.5)	57 (9.9)	3 (6.5)	1 (1.4)	0.050
Statin	1743 (100.0)	1051 (100.0)	573 (100.0)	46 (100.0)	73 (100.0)	-
Beta-blockers	1225 (70.3)	728 (45.8)	418 (72.9)	28 (60.9)	51 (69.9)	0.212
ACEI/ARB	841 (48.3)	481 (45.8)	301 (52.5)	20 (43.5)	39 (53.4)	0.045
Furosemide	114 (6.5)	53 (5.0)	46 (8.0)	2 (4.3)	13 (17.8)	< 0.001
**Outcomes**						
MACEs, n (%)	351 (20.1)	170 (16.2)	144 (25.1)	7 (15.2)	30 (41.1)	< 0.001
Cardiac death, n (%)	37 (2.1)	12 (1.1)	14 (2.5)	2 (4.3)	9 (12.3)	< 0.001
Non-fatal MI, n (%)	43 (2.5)	20 (1.9)	19 (3.3)	1 (2.2)	3 (4.1)	0.268
Non-fatal stroke, n (%)	22 (1.3)	9 (0.9)	12 (2.1)	1 (2.2)	0 (0)	0.121
Unplanned revascularization, n (%)	249 (14.3)	129 (12.3)	99 (17.3)	3 (6.5)	18 (24.7)	0.003

The p-value represented whether the difference between the proportions or means among the four groups was significant

*Abbreviations* ACEI, angiotensin-converting enzyme inhibitor; ACS, acute coronary syndrome; ARB, angiotensin receptor blockade; CAD, coronary artery disease; COPD, chronic obstructive pulmonary disease; CV, cardiovascular; EF, ejection fraction; GRACE, the Global Registry of Acute Coronary Events; HDL-C, high-density lipoprotein cholesterol; LDL-C, low-density lipoprotein cholesterol; LVSD, left ventricular systolic dysfunction; MACEs, major adverse cardiac events; NRI, Nutrition risk index; NSTEMI, non-ST-elevation myocardial infarction; PAD, peripheral artery disease; PCI, percutaneous coronary intervention; STEMI, ST-elevation myocardial infarction; TC, total cholesterol; TG, triglycerides; WBC, white blood cell

The adjusted hazard ratio and 95%CI of inflammation or malnutrition on clinical outcomes are shown in Table 2; the association between the covariates included in the multivariable model and MACEs was displayed in Supplementary Table 1. Malnutrition showed a significant correlation to the subsequent cardiac death, unplanned revascularization, and overall MACEs; the high-inflamed status was related to the increased MACEs, mainly driven by the incidence of unplanned revascularization.

**Table 2:** Predictive value of malnutrition or inflammation for outcomes in univariate and multivariate proportional hazards regression analyses

	Crude HR (95% CI)	*p*-value	Adjusted HR (95% CI)	*P* value
**Malnutrition** ^**a**^				
MACEs	1.790 (1.273, 2.516)	< 0.001	1.629 (1.109, 2.393)	0.013
Cardiac death	6.158 (3.043, 12.465)	< 0.001	4.014 (1.603, 10.049)	0.003
Non-fatal MI	1.260 (0.453, 3.502)	0.658	1.127 (0.346, 3.676)	0.843
Non-fatal stroke	1.995 (0.597, 6.666)	0.262	0.413 (0.088, 1.931)	0.261
Unplanned revascularization	1.429 (0.948, 2.153)	0.088	1.657 (1.064, 2.582)	0.025
**Hs-CRP > 2.0mg/L** ^**b**^				
MACEs	1.775 (1.440, 2.189)	< 0.001	1.331 (1.055, 1.678)	0.016
Cardiac death	2.819 (1.451, 5.478)	0.002	1.335 (0.617, 2.886)	0.463
Non-fatal MI	1.935 (1.104, 3.391)	0.021	1.425 (0.741, 2.739)	0.288
Non-fatal stroke	2.582 (1.160, 5.748)	0.020	1.019 (0.948, 1.095)	0.615
Unplanned revascularization	1.624 (1.290, 2.045)	< 0.001	1.336 (1.037, 1.720)	0.025

malnutrition was regarded as a categorial variable classified by the NRI. In multivariate analysis, the NRI index was adjusted for age, sex, heart rate, systolic blood pressure, diastolic blood pressure, hypertension, diabetes, PAD, OMI, prior PCI, prior stroke, anemia, TC, LDL-C, hs-CRP, CrCl, FPG, HbA1C, LVSD, multivessel coronary artery disease, SYNTAX score, complete revascularization, discharged with dual antiplatelet therapy, discharged with statin, and discharged with beta-blockers

Hs-CRP determined the high-inflamed status over the 2.2 mg/L cut-off value. In multivariate analysis, the hs-CRP was adjusted for age, sex, heart rate, systolic blood pressure, diastolic blood pressure, hypertension, diabetes, PAD, OMI, prior PCI, prior stroke, anemia, TC, LDL-C, albumin, CrCl, FPG, HbA1C, LVSD, multivessel coronary artery disease, SYNTAX score, complete revascularization, discharged with dual antiplatelet therapy, discharged with statin, and discharged with beta-blockers

*Abbreviations* BNP, B-type natriuretic peptide; CAD, coronary artery disease; CI, confidence interval; CrCl, creatinine clearance rate; FPG, fasting plasma glucose; HbA1C, glycated hemoglobin A1c; HDL-C, high-density lipoprotein cholesterol; HR, hazard ratio; hs-CRP, high-sensitivity C-reactive protein; LDL-C, low-density lipoprotein cholesterol; LVSD, left ventricular systolic dysfunction; MACEs, major adverse cardiac events; MI, myocardial infarction; NLR, neutrophil-lymphocyte ratio; NRI, Nutrition risk index; OMI, old myocardial infarction; PAD, peripheral artery disease; PCI, percutaneous coronary intervention; TC, total cholesterol; TG, triglycerides; WBC, white blood cell

**Fig. 2:**
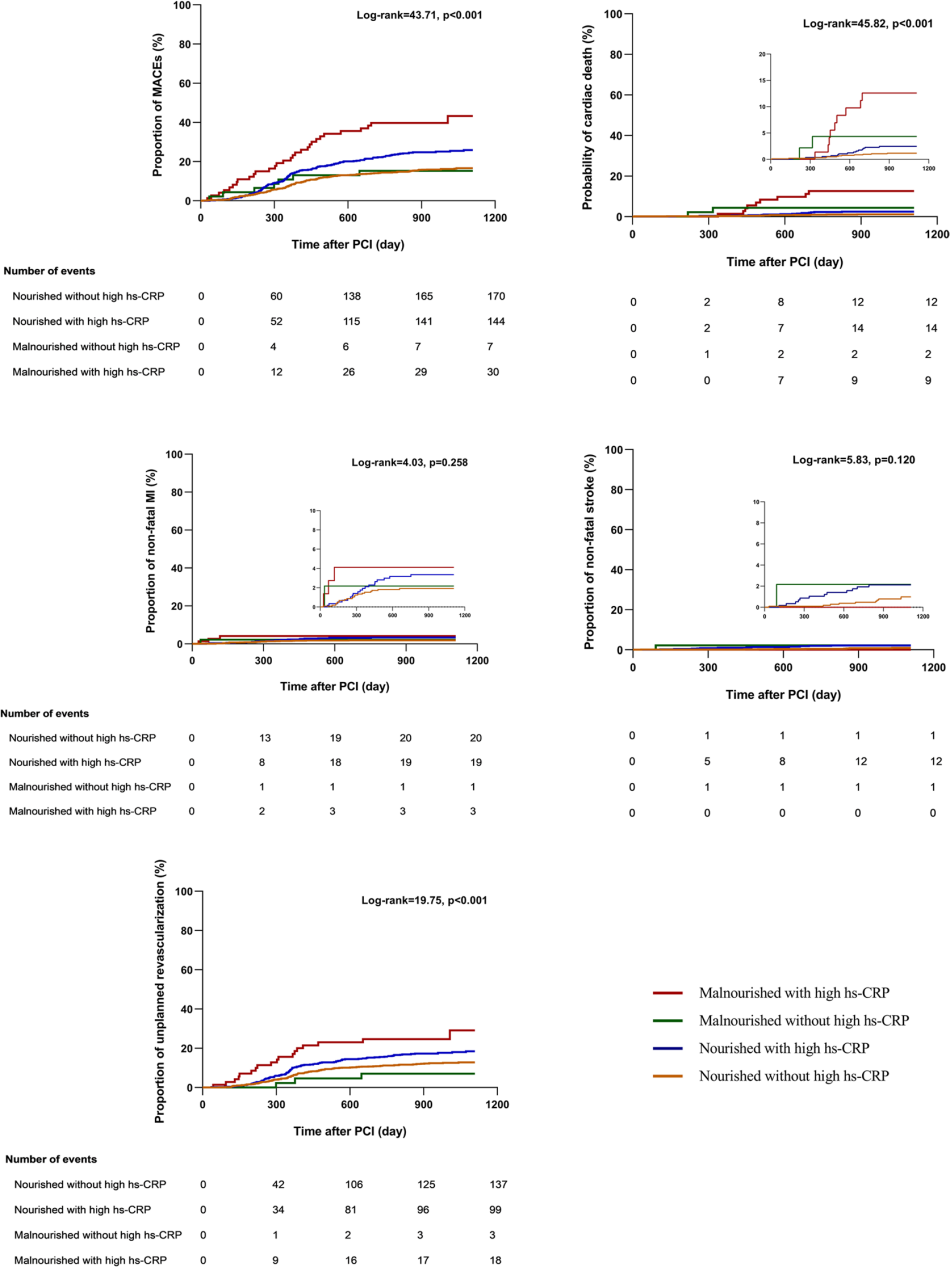
Kaplan-Meier survival curves for MACEs and each component up to 3 years *Abbreviations* hs-CRP, high-sensitivity C-reactive protein; MACEs, major adverse cardiac events; MI, myocardial infarction; PCI, percutaneous coronary intervention

Kaplan-Meier survival analyses showed that malnourished patients with elevated hs-CRP levels exhibited the most unfavorable prognosis in terms of MACEs, cardiac death, and unplanned revascularization. However, no difference among the four groups was found in non-fatal MI or non-fatal stroke (Fig. 2). Using nourished without the elevated hs-CRP group as the reference, malnourished patients with elevated hs-CRP showed significantly higher MACEs, cardiac death, and unplanned revascularization in multivariate analyses (all *P* < 0.05); the nourished with elevated hs-CRP group presented with increased unplanned revascularization (*P* = 0.005). Interestingly, patients in the malnourished group without elevated hs-CRP exhibited no significant difference in affecting MACEs and each component compared with the reference group (Table 3). We further conducted the subgroup analyses evaluating the predictive value of nutrition-inflammation status in patients stratified by ACS type, and no evidence of subgroup interactions was observed between patients diagnosed with AMI or unstable angina (Supplementary Table 3).

**Table 3:** Predictive value of each group on outcomes in multivariate proportional hazards regression analyses ^a^

	Nourished and noninflamed (*n* = 1051)	Nourished and high-inflamed (*n* = 573)	Malnourished and noninflamed (*n* = 46)	Malnourished and high-inflamed (*n* = 73)
**MACEs**				
Adjusted HR (95% CI)	ref	1.302 (0.591, 2.866)	1.237 (0.533, 2.858)	2.446 (1.464, 4.089)
*P* value		0.512	0.085	< 0.001
**Cardiac death**				
Adjusted HR (95% CI)	ref	1.456 (0.612, 3.462)	1.474 (0.579, 3.750)	25.740 (6.337, 104.554)
*P* value		0.395	0.416	< 0.001
**Non-fatal MI**				
Adjusted HR (95% CI)	ref	1.156 (0.495, 2.702)	1.289 (0.158, 10.521)	2.186 (0.617, 7.746)
*P* value		0.689	0.813	0.226
**Non-fatal stroke**				
Adjusted HR (95% CI)	ref	0.764 (0.211, 2.762)	2.588 (0.271, 24.708)	- ^b^
*P* value		0.682	0.409	- ^b^
**Unplanned revascularization**				
Adjusted HR (95% CI)	ref	1.462 (1.120, 1.909)	0.489 (0.153, 1.566)	2.349 (1.321, 4.177)
*P* value		0.005	0.228	0.004

Adjusted for age, sex, heart rate, systolic blood pressure, diastolic blood pressure, hypertension, diabetes, PAD, OMI, prior PCI, prior stroke, anemia, TC, LDL-C, CrCl, FPG, HbA1C, LVSD, multivessel coronary artery disease, SYNTAX score, complete revascularization, discharged with dual antiplatelet therapy, discharged with statin, and discharged with beta-blockers

None case of non-fatal stroke was reported in the malnourished with elevated hs-CRP group

*Abbreviations* BNP, B-type natriuretic peptide; CAD, coronary artery disease; CI, confidence interval; CrCl, creatinine clearance rate; EF, ejection fraction; FPG, fasting plasma glucose; HbA1C, glycated hemoglobin A1c; HDL-C, high-density lipoprotein cholesterol; HR, hazard ratio; hs-CRP, high-sensitivity C-reactive protein; LDL-C, low-density lipoprotein cholesterol; MACEs, major adverse cardiac events; MI, myocardial infarction; NLR, neutrophil-lymphocyte ratio; NRI, Nutrition risk index; PAD, peripheral artery disease; OMI, old myocardial infarction; PCI, percutaneous coronary intervention; ref, reference; TC, total cholesterol; TG, triglycerides; WBC, white blood cell

We further conducted stratified analyses within subgroups and calculated interaction statistics to estimate the interplay between baseline malnutrition and inflammation regarding MACEs. As shown in Table 4, the *P*-value for interaction between nutritional status (nourished vs. malnourished) and hs-CRP levels (≤ 2 mg/L vs. >2 mg/L) on MACEs was 0.248; when including hs-CRP in the model as a continuous variable, it remained a risk enhancer among the malnourished (*P* = 0.003) but not the nourished (*P* = 0.620), with evidence for interaction (P for interaction = 0.001). Simultaneously, as it was included in the model as a continuous variable, the NRI score shared a significant correlation with MACEs in high-inflamed patients (*P* = 0.003) while failing to present it in those without (*P* = 0.869), with evidence for interaction (P for interaction = 0.005) (Table 5). When hs-CRP and malnutrition were both included in the model as continuous variables, there was evidence for effect modification between each other on the association of MACEs (*P* for interaction = 0.046).

**Table 4:** Interplay of inflammation or malnutrition on MACEs in multivariable analyses ^a^. Examining the effect of inflammation on MACEs in patients stratified by nutritional status

Examined variable	Nourished subpopulation			Malnourished subpopulation			*P* for interaction
	Events (%)	Adjusted HR (95% CI)	*P* value	Events (%)	Adjusted HR (95% CI)	*P* value	
Hs-CRP > 2.0 mg/L, categorically	324 (20.0)	1.237 (0.971, 1.576)	0.085	37 (31.1)	4.858 (1.353, 17.440)	0.015	0.248
Hs-CRP level, continuously	324 (20.0)	1.006 (0.982, 1.030)	0.620	37 (31.1)	1.084 (1.027, 1.143)	0.003	0.001

**Table 5:** Examining the effect of malnutrition on MACEs in patients stratified by inflammatory status

Examined variable	Hs-CRP ≤ 2.2 mg/L subpopulation			Hs-CRP > 2.2 mg/L subpopulation			*P* for interaction
	Events (%)	Adjusted HR (95% CI)	*P* value	Events (%)	Adjusted HR (95% CI)	*P* value	
Malnourished status, categorically	177 (16.1)	1.287 (0.587, 2.832)	0.531	174 (26.9)	1.927 (1.232, 3.014)	0.004	0.248
The NRI score, continuously	177 (16.1)	1.002 (0.997, 1.028)	0.869	174 (26.9)	0.965 (0.943, 0.988)	0.003	0.005

Adjusted for age, sex, heart rate, systolic blood pressure, diastolic blood pressure, hypertension, diabetes, PAD, OMI, prior PCI, anemia, WBC, TC, LDL-C, BNP, CrCl, uric acid, FPG, HbA1C, LVSD, multivessel coronary artery disease, SYNTAX score, complete revascularization, discharged with dual antiplatelet therapy, discharged with statin, and discharged with beta-blockers

*Abbreviations* BNP, B-type natriuretic peptide; CAD, coronary artery disease; CI, confidence interval; CrCl, creatinine clearance rate; EF, ejection fraction; FPG, fasting plasma glucose; HbA1C, glycated hemoglobin A1c; HDL-C, high-density lipoprotein cholesterol; HR, hazard ratio; hs-CRP, high-sensitivity C-reactive protein; LDL-C, low-density lipoprotein cholesterol; MACEs, major adverse cardiac events; MI, myocardial infarction; NLR, neutrophil-lymphocyte ratio; NRI, Nutrition risk index; OMI, old myocardial infarction; PAD, peripheral artery disease; PCI, percutaneous coronary intervention; ref, reference; TC, total cholesterol; TG, triglycerides; WBC, white blood cell

Finally, the incremental predictive effect of hs-CRP or NRI beyond the established GRACE risk score was evaluated. As adding each variate into the GRACE model significantly increased the original predictive value on MACEs or cardiac death, a synergistic effect showed when combining both hs-CRP and NRI with the GRACE model (Table 6 and Supplementary Table 4).

**Table 6:** Incremental effect of inflammation or malnutrition beyond the GRACE risk model to predict MACEs and cardiac death

Model	C-index (95%CI)	*P* value	Net reclassification improvement (95%CI)	*P* value	Integrated discrimination Improvement (95%CI)	*P* value
**MACEs**						
GRACE ^a^	0.538 (0.514, 0.562)	0.003	ref		ref	
GRACE + NRI ^b^	0.554 (0.530, 0.577)	< 0.001	0.128 (0.011, 0.245)	0.032	0.005 (0.002, 0.009)	0.004
GRACE + hs-CRP ^c^	0.590 (0.566, 0.613)	< 0.001	0.196 (0.088, 0.303)	< 0.001	0.006 (0.002, 0.010)	0.005
GRACE + NRI + hs-CRP	0.594 (0.570, 0.617)	< 0.001	0.210 (0.094, 0.326)	< 0.001	0.010 (0.005, 0.016)	< 0.001
**Cardiac death**						
GRACE	0.699 (0.677, 0.721)	< 0.001	ref		ref	
GRACE + NRI	0.733 (0.712, 0.754)	< 0.001	0.629 (0.318, 0.940)	< 0.001	0.020 (0.007, 0.033)	0.002
GRACE + hs-CRP	0.702 (0.680, 0.723)	< 0.001	0.369 (0.050, 0.688)	0.023	0.006 (0.001, 0.012)	0.049
GRACE + NRI ^b^ + hs-CRP ^c^	0.738 (0.717, 0.759)	< 0.001	0.666 (0.361, 0.971)	< 0.001	0.023 (0.009, 0.037)	0.002

The eight variables that constitute the GRACE risk score are age, history of heart failure, history of acute MI, heart rate and systolic blood pressure at admission, ST-segment depression, serum creatinine at admission, and elevated myocardial necrosis markers or enzymes

NRI was introduced to the GRACE model as a dichotomous variable

Hs-CPR was introduced to the GRACE model as a dichotomous variable

*Abbreviations* C-index, concordance index; CI, confidence interval; GRACE, the Global Registry of Acute Coronary Events; hs-CRP, high-sensitivity C-reactive protein; MACEs, major adverse cardiac events; NRI, Nutrition risk index; ref, reference

## Discussion

This study of retrospective data is the first to highlight the importance of an easily overlooked population of ACS patients, who suffered a double burden of malnutrition and high-inflamed status. We validated that among ACS patients, malnutrition combined with inflammation is associated with a significantly increased risk of long-term cardiac outcomes and may interplay by amplifying the harmful effects of each other, adding both of which into the GRACE risk score would provide an increment predictive value.

Statistics about the prevalence of malnutrition among ACS patients varied across these years’ studies. A large cohort involving 10,161 AMI patients who underwent PCI showed that 11% were at risk of malnutrition, as determined by GNRI [19]. Komici et al. evaluated the nutritional status of 174 elderly AMI patients by Mini Nutritional Assessment (MNA), and 12% were considered malnourished [20]; Tonet et al. reported 4% of elderly ACS patients undernourished using the MNA–Short Form [21]. Data from 5,062 ACS patients suggested that malnutrition prevailed in over 50% of cases based on the controlling nutritional status (CONUT) score, NRI, or prognostic nutritional index (PNI) [22]. Recently, Kong et al. observed 1,829 AMI patients, 76.5%, 61.8%, and 39.3% of whom were malnourished according to the CONUT score, NRI, and PNI, respectively [3]. These variations reflect the necessity and importance of setting universal, standardized, and pragmatic nutritional evaluation criteria to facilitate risk stratification and management. In 2018, the American Society for Parenteral and Enteral Nutrition (ASPEN) led the launching of the GLIM criteria as a consensus-based criterion of malnutrition [23]; however, the demand for body composition measuring techniques may hinder its widely applied in a general setting, especially a coronary care unit. Current frequently used screening tools have exhibited various discrimination abilities. However, some may not be suitable, considering the particularity of the ACS population. To be specific, one of the items of the criterion of the CONUT score is total cholesterol, which could be strongly perturbed by patients who have received lipid-lowering therapies. Thus, there’s an unmet need to establish an ACS-suitable nutritional evaluation method to facilitate clinical evaluation and prevention systems.

In our cohort, older adults, females, and leaner people were found more frequently in the malnourished group, and they also displayed lower cholesterol levels; these clinical characteristics are consistent with the former studies [3, 22]. One of the notable observations of our study is that a heavy burden of systemic inflammation marked by elevated hs-CRP prevailed in malnourished ACS patients by over 60%. Inflammation is thought to be a key driver for sarcopenia elicited by an underlying illness, leading to disease-related malnutrition and increased mortality [24, 25]. CAD has been recognized as not only a lipid metabolic disorder but also an inflammation-mediated systemic disease, with the pro- and anti-inflammatory reactions interacting to exacerbate or ameliorate the atherosclerotic process and causing catabolic activity, energy expenditure, and function loss. One of the primary underlying mechanisms is the circulating cytokines released from the systemic inflammatory response (i.e., interleukin 6, interleukin 1β, and tumor necrosis factors), triggering mechanisms that contribute to the pathogenesis of malnutrition, including brain circuits disorder, protein degradation, and neuroendocrine disturbance, ultimately causing disease-related anorexia, sarcopenia, cognitive decline, and frailty [11, 12, 26, 27]. In 2012, ASPEN and the Academy of Nutrition and Dietetics launched a consensus statement emphasizing the need to identify inflammation early in the diagnostic procedure of malnutrition to determine its etiology and guide treatment [28]. In a traumatic and acute stress-causing event like ACS, albumin serves as a practical plasma biomarker that can be used to indicate and monitor catabolic metabolism and systemic inflammatory response to reflect disease severity; hypoalbuminemia was also one of the most striking features of patients with MACEs in our cohort.

Nutritional status influences the body’s inflammatory response, and consistent nutritional management is crucial for patients with critical illness to recover from inflammatory injury [27, 29]. Although the prevalence of malnutrition exhibited significant discrepancies, its association with poorer outcomes remains clear and sound [19–22, 30, 31]. However, the importance of screening and monitoring for malnutrition in patients with ACS hasn’t drawn enough concern from clinical guidelines and practice [32, 33]. The subgroup analysis of the present study suggested the bidirectional association between inflammation and malnutrition; however, the interaction differed when handling inflammation and malnutrition as binary or continuous values, which indicates that the stratification standards to define high inflammation or malnutrition seemingly may affect the results. In this scenario, studies with larger scale and ACS-suitable criteria for stratification seemed indispensable to facilitate the understanding of the interplay between inflammation and malnutrition. Given the existing results, we could speculate that patients with worse nutritional conditions may urgently need deactivating inflammation, and patients with higher hs-CRP would benefit more from nutritional support. Previous studies among hospitalized patients showed that baseline inflammatory status was associated with their response to nutritional therapy and that nutritional support alone is inadequate to prevent muscle loss during severe, sustained, or repeated bouts of inflammation [34, 35], which emphasizes the importance of considering the double burden of inflammation and malnutrition in disease assessment, intervention, and monitoring. Regarding ACS patients, there is still a lack of evidence investigating the treatment response to nutritional support or anti-inflammatory therapy among subgroups with different degrees of inflammation or malnutrition.

To the best of our knowledge, the present study is the first analysis to investigate the interplay between inflammation and malnutrition in affecting cardiac outcomes among ACS patients, which would provide preliminary implications for future research. We are also aware of several limitations. First, as a retrospective study, we couldn’t conclude direct causality, account for other potential confounding factors, and were more susceptible to selection, information, and recall bias. Second, the proportion of AMI patients (26%) in our ACS cohort was lower than the statistics from large-scale Chinese registries [36, 37], which may be attributed to the limited sample size; thus, the study results do not necessarily reflect the outcome of ACS patients treated according to a modern standard. Third, the diagnostic accuracy of NRI among ACS patients was yet to be confirmed, and the GLIM criteria were regrettably not used due to a lack of indispensable information; these existing problems need to be addressed in future research works as we move towards a global definition of malnutrition. Fourth, the serum hs-CRP, albumin, and body weight were measured only once at admission and are subject to measurement error and physiologic variability; the dynamic and long-term monitoring of inflammatory and nutritional status changes would provide valuable and indispensable information. Fifth, as it was a single-center study based on Chinese people, the results should be carefully interpreted and extended to other ethnicities.

## Conclusions

Among patients with ACS undergoing PCI, the double burden of inflammation and malnutrition was not uncommon and strongly associated with long-term cardiac outcomes in a synergistic manner. Identifying patients’ inflammatory and nutritional status will better facilitate the modern individualized care of ACS. Future prospective studies are needed to evaluate whether the heterogeneity in the therapeutic effect of nutritional support or anti-inflammatory interventions exists in patients with different inflammatory or nutritional burdens.

### Electronic supplementary material

Below is the link to the electronic supplementary material.

Supplementary Material 1

## Data Availability

The data that support the findings of this study are not openly available due to reasons of sensitivity and are available from the corresponding author upon reasonable request. Data are located in controlled access data storage at Beijing Anzhen Hospital, China.

## Abbreviations

ACS: Acute coronary syndrome
CONUT: Controlling nutritional status
CVD: Cardiovascular diseases
GLIM: Global Leadership Initiative on Malnutrition
GNRI: Geriatric nutritional risk index
GRACE: Global Registry of Acute Coronary Events
Hs-CRP: High-sensitivity C-reactive protein
MACEs: Major adverse cardiovascular events
PCI: Percutaneous coronary intervention
PNI: Prognostic nutritional index
