# Supplementary material for: Combined effect of inflammation and malnutrition for long-term prognosis in patients with acute coronary syndrome undergoing percutaneous coronary intervention: a cohort study
Source: BMC Cardiovasc Disord. 2024 Jun 17;24:306. doi: 10.1186/s12872-024-03951-7 (PMC11181542; doi:10.1186/s12872-024-03951-7)
Supplement: Supplementary file 1 — Supplementary Material 1 [file 12872_2024_3951_MOESM1_ESM.docx]

**Table S1 Univariate Cox regression analysis for MACEs predictors.**

| Variable | Univariate analysis | |
| --- | --- | --- |
|  | HR (95% CI) | p-value |
| Age | 1.006 (0.996, 1.017) | 0.213 |
| Sex, male | 1.072 (0.833, 1.379) | 0.589 |
| Body mass index | 0.971 (0.937, 1.006) | 0.101 |
| Heart rate | 1.032 (1.021, 1.042) | <0.001 |
| Systolic blood pressure | 1.007 (1.001, 1.013) | 0.024 |
| Diastolic blood pressure | 0.973 (0.963, 0.983) | <0.001 |
| Hypertension | 1.043 (0.838, 1.298) | 0.708 |
| Diabetes | 1.560 (1.264, 1.926) | <0.001 |
| Peripheral artery disease | 2.815 (2.183, 3.630) | <0.001 |
| Old myocardial infarction | 1.551 (1.223, 1.968) | <0.001 |
| Prior PCI | 1.584 (1.252, 2.003) | <0.001 |
| Prior stroke | 0.994 (0.633, 1.561) | 0.980 |
| Anemia | 2.431 (1.593, 3.710) | <0.001 |
| Total cholesterol | 1.151 (1.041, 1.272) | 0.006 |
| Low-density lipoprotein cholesterol | 1.189 (1.053, 1.342) | 0.005 |
| B-type natriuretic peptide | 1.001 (1.000, 1.001) | 0.034 |
| Creatinine clearance rate | 0.993 (0.990, 0.997) | <0.001 |
| Fasting plasma glucose | 1.010 (1.008, 1.013) | <0.001 |
| Glycated hemoglobin A1c | 1.177 (1.095, 1.264) | <0.001 |
| LVSD (EF < 50%) | 1.569 (1.079, 2.281) | 0.018 |
| Multivessel coronary artery disease | 2.160 (1.494, 3.123) | <0.001 |
| SYNTAX score | 1.036 (1.027, 1.045) | <0.001 |
| Complete revascularization | 0.429 (0.347, 0.530) | <0.001 |
| Dual antiplatelet therapy | 0.241 (0.128, 0.452) | <0.001 |
| Statin ^a^ | - | - |
| Beta-blockers | 0.782 (0.628, 0.975) | 0.029 |

1. All of the study participants were treated with statin on discharge.

Abbreviations: ACEI, angiotensin-converting enzyme inhibitor; ACS, acute coronary syndrome; ARB, angiotensin receptor blockade; CAD, coronary artery disease; CI, confidence interval; COPD, chronic obstructive pulmonary disease; CV, cardiovascular; EF, ejection fraction; GRACE, the Global Registry of Acute Coronary Events; HR, hazard ratio; LVSD, left ventricular systolic dysfunction; MACEs, major adverse cardiac events; NSTEMI, non-ST-elevation myocardial infarction; PCI, percutaneous coronary intervention; SMuRFs, standard modifiable CV risk factors; STEMI, ST-elevation myocardial infarction; SYNTAX, Synergy Between Percutaneous Coronary Intervention with Taxus and Cardiac Surgery.

**Table S2 Baseline clinical characteristics of patients stratified by the occurrence of MACEs.**

| Variable | Total population  （n=1743） | Without MACEs  （n=1392） | With MACEs  （n=351） | p-value |
| --- | --- | --- | --- | --- |
| **Demography and anthropometric data** |  |  |  |  |
| Age, years | 60 (53, 67) | 61 (53, 67) | 62 (53, 68) | 0.318 |
| Sex, male, n (%) | 1333 (76.5) | 1060 (76.1) | 273 (77.8) | <0.001 |
| Body mass index, kg/m^2^ | 25.2 (23.6, 27.6) | 25.3 (23.7, 27.6) | 24.9 (23.3, 27.7) | 0.114 |
| Heart rate, bpm | 68 (62, 75) | 67 (61, 73) | 70 (64, 79) | <0.001 |
| Systolic blood pressure, mmHg | 130 (120, 140) | 130 (120, 140) | 130 (120, 140) | 0.046 |
| Pulse pressure, mmHg | 50 (44, 62) | 50 (43, 60) | 57 (48, 68) | <0.001 |
| Family history of CAD, n (%) | 554 (31.5) | 425 (30.5) | 129 (36.8) | 0.025 |
| **CV risk factors and prior CV disease** |  |  |  |  |
| Hypertension, n (%) | 1112 (63.8) | 885 (63.6) | 227 (64.7) | 0.703 |
| Diabetes, n (%) | 805 (46.2) | 609 (43.8) | 196 (55.8) | <0.001 |
| Hypercholesterolaemia, n (%) | 1396 (80.1) | 1103 (79.2) | 293 (83.5) | 0.076 |
| Smoking, n (%) | 1025 (58.8) | 812 (58.3) | 213 (60.7) | 0.424 |
| Peripheral artery disease, n (%) | 183 (10.5) | 107 (7.7) | 76 (21.7) | <0.001 |
| Old myocardial infarction, n (%) | 333 (19.1) | 241 (17.3) | 92 (26.2) | <0.001 |
| Prior PCI, n (%) | 343 (19.7) | 247 (17.7) | 96 (27.4) | <0.001 |
| Prior Cardiac arrest, n (%) | 2 (0.1) | 0 | 2 (0.6) | 0.005 |
| Atrial fibrillation/flutter, n (%) | 74 (4.2) | 56 (4.0) | 18 (5.1) | 0.359 |
| Prior stroke, n (%) | 102 (5.9) | 82 (5.9) | 20 (5.7) | 0.791 |
| **Comorbidity** |  |  |  |  |
| Anemia ^b^, n (%) | 57 (3.3) | 34 (2.4) | 23 (6.6) | <0.001 |
| COPD, n (%) | 26 (1.5) | 22 (1.6) | 4 (1.1) | 0.543 |
| Cancer history, n (%) | 13 (0.7) | 11 (0.8) | 2 (0.6) | 0.668 |
| **ACS presentation** |  |  |  |  |
| Type of ACS, n (%) |  |  |  |  |
| STEMI | 229 (13.1) | 184 (13.2) | 45 (12.8) | 0.844 |
| NSTEMI | 227 (13.0) | 175 (12.6) | 52 (14.8) | 0.265 |
| Unstable angina | 1287 (73.8) | 1033 (74.2) | 254 (72.4) | 0.482 |
| Killip class >1, n (%) | 67 (3.8) | 48 (3.4) | 19 (5.4) | 0.087 |
| The GRACE risk score, points | 98 (82, 114) | 98 (82, 112) | 99 (82, 122) | 0.048 |
| High mortality risk at six months, n (%) | 356 (20.4) | 263 (18.9) | 93 (26.5) | 0.002 |
| **Laboratory data and Echocardiographic data** | |  |  |  |
| White blood cell, 10^9^/L | 6.3 (6.3, 7.5) | 6.2 (5.3, 7.3) | 6.8 (5.7, 8.0) | <0.001 |
| High-sensitivity C-reactive protein, mg/L | 1.7 (0.7, 3.5) | 1.3 (0.6, 3.2) | 2.1 (0.9, 5.3) | <0.001 |
| Triglycerides, mg/dL | 1.5 (1.0, 2.1) | 1.4 (1.0, 2.0) | 1.6 (1.1, 2.3) | <0.001 |
| Total cholesterol, mg/dL | 4.0 (3.4, 4.8) | 4.0 (3.4, 4.7) | 4.2 (3.5, 5.0) | <0.001 |
| Low-density lipoprotein cholesterol, mg/dL | 2.3 (1.8, 3.0) | 2.3 (1.8, 2.9) | 2.5 (2.0, 3.1) | <0.001 |
| High-density lipoprotein cholesterol, mg/dL | 1.0 (0.9, 1.2) | 1.0 (0.9, 1.2) | 1.0 (0.9, 1.1) | <0.001 |
| B-type natriuretic peptide, pg/ml | 37 (21, 86) | 34 (20, 79) | 52 (27, 98) | <0.001 |
| Albumin, g/L | 42 (40, 45) | 42 (40, 45) | 41 (39, 44) | <0.001 |
| Creatinine clearance rate, ml/min | 96.7 (78.8, 116.7) | 97.2 (80.5, 117.7) | 94.0 (74.4, 113.1) | 0.007 |
| Uric acid, μmol/L | 333.2 (289.3, 397.8) | 325.4 (287.4, 392.2) | 353.6 (304.0, 409.8) | <0.001 |
| Fasting plasma glucose, mmol/L | 5.8 (5.2, 7.0) | 5.7 (5.2, 6.8) | 6.3 (5.5, 8.1) | <0.001 |
| Glycated hemoglobin A1c, % | 6.1 (5.6, 7.1) | 6.0 (5.5, 7.0) | 6.4 (5.7, 7.5) | <0.001 |
| LVSD (EF < 50%), n (%) | 106 (6.1) | 76 (5.5) | 30 (8.5) | 0.031 |
| **Angiographic data** |  |  |  |  |
| Left main coronary artery lesion, n (%) | 136 (7.8) | 103 (7.4) | 33 (9.4) | 0.211 |
| Multivessel coronary artery disease, n (%) | 1457 (83.6) | 1137 (81.7) | 320 (91.2) | <0.001 |
| Chronic total occlusion, n (%) | 367 (21.1) | 280 (20.1) | 87 (24.8) | 0.055 |
| SYNTAX score | 20 (13, 28) | 19 (12, 27) | 25 (17, 33) | <0.001 |
| Complete revascularization, n (%) | 1069 (61.3) | 918 (65.9) | 151 (43.0) | <0.001 |
| The percutaneous transfemoral route, n (%) | 27 (1.5) | 24 (1.7) | 3 (0.9) | 0.239 |
| **Medical therapy** |  |  |  |  |
| Before admission, n (%) |  |  |  |  |
| Antiplatelet agents | 1272 (73.0) | 1010 (72.6) | 254 (74.6) | 0.432 |
| Statin | 1252 (71.8) | 994 (71.4) | 258 (73.5) | 0.435 |
| On discharge, n (%) |  |  |  |  |
| Dual antiplatelet therapy | 1727 (99.1) | 1386 (99.6) | 341 (97.2) | <0.001 |
| Aspirin plus clopidogrel | 1587 (91.0) | 1279 (91.9) | 308 (87.7) | 0.015 |
| Aspirin plus ticagrelor | 140 (8.0) | 107 (7.7) | 33 (9.4) | 0.291 |
| Statin | 1743 (100.0) | 1392 (100.0) | 351 (100.0) | - |
| Beta-blockers | 1225 (70.3) | 995 (71.5) | 230 (65.5) | 0.029 |
| ACEI/ARB | 841 (48.3) | 661 (47.5) | 180 (51.3) | 0.203 |
| Furosemide | 114 (6.5) | 89 (6.4) | 25 (7.1) | 0.622 |

1. SMuRF-less represents no evidence of standard modifiable CV risk factors (SMuRFs), which include hypertension, diabetes, hypercholesterolemia, and smoking.
2. Anemia was defined as hemoglobin < 120 g/L for an adult male, and <110 g/L for an adult female.
3. The malnutrition rate was counted exclusively in patients with AMI, with a total of 456, and 97 MACEs occurred.

Abbreviations: ACEI, angiotensin-converting enzyme inhibitor; ACS, acute coronary syndrome; ARB, angiotensin receptor blockade; CAD, coronary artery disease; COPD, chronic obstructive pulmonary disease; CV, cardiovascular; EF, ejection fraction; GRACE, the Global Registry of Acute Coronary Events; LVSD, left ventricular systolic dysfunction; MACEs, major adverse cardiac events; NSTEMI, non-ST-elevation myocardial infarction; PCI, percutaneous coronary intervention; SMuRFs, standard modifiable CV risk factors; STEMI, ST-elevation myocardial infarction; SYNTAX, Synergy Between Percutaneous Coronary Intervention with Taxus and Cardiac Surgery.

**Table S3 Predictive value of each group on outcomes in multivariate proportional hazards regression analyses stratified by ACS diagnosis** ^a^**.**

|  |  | Nourished and high-inflamed vs. Nourished and noninflamed | | | |  | Malnourished and noninflamed vs. Nourished and noninflamed | | | |  | Malnourished and high-inflamed vs. Nourished and noninflamed | | | |
| --- | --- | --- | --- | --- | --- | --- | --- | --- | --- | --- | --- | --- | --- | --- | --- |
|  |  | n/N, % | Adjusted HR (95% CI) | *P*-value | *P* _interaction_ |  | n/N, % | Adjusted HR (95% CI) | *P*-value | *P* _interaction_ |  | n/N, % | Adjusted HR (95% CI) | *P*-value | *P* _interaction_ |
| **MACEs** |  |  |  |  |  |  |  |  |  |  |  |  |  |  |  |
| Overall patients |  | 314/1624, 19.3 | 1.302 (0.591, 2.866) | 0.512 |  |  | 177/1098, 16.1 | 1.237 (0.533, 2.858) | 0.085 |  |  | 200/1124, 17.8 | 2.446 (1.464, 4.089) | <0.001 |  |
| ACS-stratified |  |  |  |  |  |  |  |  |  |  |  |  |  |  |  |
| AMI |  | 79/403, 19.6 | 1.791 (1.017, 3.153) | 0.043 | 0.559 |  | 30/206, 14.6 | 0.792 (0.090, 6.955) | 0.834 | 0.604 |  | 46/235, 19.6 | 3.311 (1.047, 10.474) | 0.042 | 0.771 |
| UA |  | 235/1221, 19.2 | 1.230 (0.932, 1.624) | 0.144 |  |  | 147/891, 16.5 | 1.535 (0.971, 1.577) | 0.622 |  |  | 154/889, 17.3 | 2.310 (1.194, 4.471) | 0.013 |  |
| **Cardiac death** |  |  |  |  |  |  |  |  |  |  |  |  |  |  |  |
| Overall patients |  | 26/1624, 1.6 | 1.456 (0.612, 3.462) | 0.395 |  |  | 14/1098, 1.3 | 1.474 (0.579, 3.750) | 0.416 |  |  | 21/1124, 1.9 | 25.740 (6.337, 104.554) | <0.001 |  |
| ACS-stratified |  |  |  |  |  |  |  |  |  |  |  |  |  |  |  |
| AMI |  | 9/403, 2.2 | 1.585 (0.027, 61.746) | 0.899 | 0.354 |  | 2/206, 1.0 | 1.037 (0.007, 1.416) | 0.959 | 0.989 |  | 7/235, 3.0 | 0.972 (0.007, 1.416) | 0.873 | 0.564 |
| UA |  | 17/1221, 1.4 | 0.885 (0.286, 2.737) | 0.832 |  |  | 12/891, 1.3 | 6.594 (0.899, 48.350) | 0.064 |  |  | 14/889, 1.6 | 43.927 (8.925, 166.312) | <0.001 |  |
| **Non-fatal MI** |  |  |  |  |  |  |  |  |  |  |  |  |  |  |  |
| Overall patients |  | 39/1624, 2.4 | 1.156 (0.495, 2.702) | 0.689 |  |  | 21/1098, 1.9 | 1.289 (0.158, 10.521) | 0.813 |  |  | 23/1124, 2.0 | 2.186 (0.617, 7.746) | 0.226 |  |
| ACS-stratified |  |  |  |  |  |  |  |  |  |  |  |  |  |  |  |
| AMI |  | 11/403, 2.7 | 0.187 (0.008, 4.229) | 0.292 | 0.023 |  | 7/206, 3.4 | 0.431 (0.005, 3.467) | 0.770 | 0.962 |  | 9/235, 3.8 | 0.284 (0.012, 3.467) | 0.770 | 0.939 |
| UA |  | 28/1221, 2.3 | 1.157 (0.576, 2.361) | 0.738 |  |  | 14/891, 1.6 | 1.592 (0.576, 12.361) | 0.987 |  |  | 14/889, 1.6 | 3.592 (0.756, 14.334) | 0.993 |  |
| **Non-fatal stroke** |  |  |  |  |  |  |  |  |  |  |  |  |  |  |  |
| Overall patients |  | 21/1624, 1.3 | 0.764 (0.211, 2.762) | 0.682 |  |  | 10/1098, 1.0 | 2.588 (0.271, 24.708) | 0.409 |  |  | 9/1124, 0.8 | - ^b^ | - |  |
| ACS-stratified |  |  |  |  |  |  |  |  |  |  |  |  |  |  |  |
| AMI |  | 4/403, 1.0 | 0.187 (0.008, 1.229) | 0.292 | 0.864 |  | 1/206, 0.5 | - | 1.000 | 0.933 |  | 1/235, 0.4 | - | - | - |
| UA |  | 17/1221, 1.4 | 2.225 (0.923, 5.363) | 0.075 |  |  | 9/891, 1.0 | 10.269 (0.581, 58.442) | 0.112 |  |  | 8/889, 0.9 | - | - |  |
| **Unplanned revascularization** |  |  |  |  |  |  |  |  |  |  |  |  |  |  |  |
| Overall patients |  | 228/1624, 14.0 | 1.462 (1.120, 1.909) | 0.005 |  |  | 132/1098, 12.0 | 0.489 (0.153, 1.566) | 0.228 |  |  | 147/1124, 13.1 | 2.349 (1.321, 4.177) | 0.004 |  |
| ACS-stratified |  |  |  |  |  |  |  |  |  |  |  |  |  |  |  |
| AMI |  | 55/403, 13.6 | 1.880 (0.953, 3.710) | 0.069 | 0.719 |  | 20/206, 9.7 | 0.201 (0.024, 1.021) | 0.673 | 0.953 |  | 29/235, 12.3 | 3.344 (0.519,10.586) | 0.268 | 0.509 |
| UA |  | 173/1221, 14.2 | 1.191 (0.860, 1.649) | 0.292 |  |  | 112/891, 12.6 | 0.798 (0.249, 2.559) | 0.705 |  |  | 118/889, 13.3 | 2.033 (0.914, 4.522) | 0.082 |  |

1. Adjusted for age, sex, heart rate, systolic blood pressure, diastolic blood pressure, hypertension, diabetes, PAD, OMI, prior PCI, prior stroke, anemia, TC, LDL-C, CrCl, FPG, HbA1C, LVSD, multivessel coronary artery disease, SYNTAX score, complete revascularization, discharged with dual antiplatelet therapy, discharged with statin, and discharged with beta-blockers.
2. None case of non-fatal stroke was reported in the malnourished and high-inflamed group.

Abbreviations: ACS, acute coronary syndrome; AMI, acute myocardial infarction; BNP, B-type natriuretic peptide; CAD, coronary artery disease; CI, confidence interval; CrCl, creatinine clearance rate; EF, ejection fraction; FPG, fasting plasma glucose; HbA1C, glycated hemoglobin A1c; HDL-C, high-density lipoprotein cholesterol; HR, hazard ratio; hs-CRP, high-sensitivity C-reactive protein; LDL-C, low-density lipoprotein cholesterol; MACEs, major adverse cardiac events; MI, myocardial infarction; NLR, neutrophil-lymphocyte ratio; NRI, Nutrition risk index; PAD, peripheral artery disease; OMI, old myocardial infarction; PCI, percutaneous coronary intervention; ref, reference; TC, total cholesterol; TG, triglycerides; UA, unstable angina; WBC, white blood cell.

**Table S4 Incremental effect of inflammation or malnutrition beyond the GRACE risk model** **to predict MACEs and cardiac death.**

| Model | C-index (95%CI) | *P* value | Net reclassification  improvement (95%CI) | *P* value | Integrated discrimination  Improvement (95%CI) | *P* value |
| --- | --- | --- | --- | --- | --- | --- |
| **MACEs** |  |  |  |  |  |  |
| GRACE ^a^ | 0.538 (0.514, 0.562) | 0.003 | ref |  | ref |  |
| GRACE + NRI ^b^ | 0.565 (0.541, 0.588) | <0.001 | 0.134 (0.012, 0.249) | 0.023 | 0.005 (0.002, 0.010) | 0.003 |
| GRACE + hs-CRP ^c^ | 0.568 (0.545, 0.592) | <0.001 | 0.160 (0.056, 0.298) | 0.015 | 0.006 (0.002, 0.010) | <0.001 |
| GRACE + NRI ^b^ + hs-CRP ^c^ | 0.577 (0.554, 0.600) | <0.001 | 0.176 (0.065, 0.303) | <0.001 | 0.006 (0.002, 0.012) | <0.001 |
| **Cardiac death** |  |  |  |  |  |  |
| GRACE ^a^ | 0.699 (0.677, 0.721) | <0.001 | ref |  | ref |  |
| GRACE + NRI ^b^ | 0.786 (0.766, 0.805) | <0.001 | 0.702 (0.421, 0.974) | <0.001 | 0.026 (0.009, 0.040) | <0.001 |
| GRACE + hs-CRP ^c^ | 0.739 (0.718, 0.760) | <0.001 | 0.631 (0.321, 0.942) | <0.001 | 0.020 (0.009, 0.034) | <0.001 |
| GRACE + NRI ^b^ + hs-CRP ^c^ | 0.785 (0.765, 0.804) | <0.001 | 0.697 (0.357, 0.930) | <0.001 | 0.034 (0.010, 0.042) | <0.001 |

1. The eight variables that constitute the GRACE risk score are age, history of heart failure, history of acute MI, heart rate and systolic blood pressure at admission, ST-segment depression, serum creatinine at admission, and elevated myocardial necrosis markers or enzymes.
2. NRI was introduced to the GRACE model as a continuous variable.
3. Hs-CPR was introduced to the GRACE model as a continuous variable.

Abbreviations: C-index, concordance index; CI, confidence interval; GRACE, the Global Registry of Acute Coronary Events; hs-CRP, high-sensitivity C-reactive protein; MACEs, major adverse cardiac events; NRI, Nutrition risk index; ref, reference.
